# Acute effects of Zamzam water on blood pressure and heart rate variability

**DOI:** 10.12669/pjms.36.4.1755

**Published:** 2020

**Authors:** Rabia Latif, Farrukh Majeed, Ahmed Al Sunni, Rahmah Mohammed K ALamrie, Shaykhah Nasser AlNaimi

**Affiliations:** 1 Rabia Latif, PhD. Associate Professor, Department of Physiology, College of Medicine, Imam Abdulrahman Bin Faisal University, Dammam, Saudi Arabia; 2 Farrukh Majeed, FCPS. Professor, Department of Physiology, Al Tibri Medical College, Karachi, Pakistan; 3 Ahmed Al Sunni, PhD. Associate Professor, Department of Physiology, College of Medicine, Imam Abdulrahman Bin Faisal University, Dammam, Saudi Arabia; 4 Rahmah Mohammed K ALamrie, Medical Student, College of Medicine, Imam Abdulrahman Bin Faisal University, Dammam, Saudi Arabia; 5 Shaykhah Nasser AlNaimi, Medical Student, College of Medicine, Imam Abdulrahman Bin Faisal University, Dammam, Saudi Arabia

**Keywords:** Heart rate variability, RMSSD, SDRR, Heart rate, Mineral water, Zamzam water

## Abstract

**Objective:**

There is a lack of studies exploring the effects of Zamzam water on human physiology. The present study determined the effects of Zamzam water on blood pressure and heart rate variability (HRV).

**Methods:**

This comparative interventional study was conducted at the Department of Physiology, of our university in March 2018. A total number of 97 female subjects drank 500 ml of either Zamzam water or mineral water in one minute. Finometer Pro and PowerLab (ADInstruments^R^) with ECG electrodes through bioamplifier and attached finger pulse transducer were used to collect data at the baseline (for five minutes), during (for one minute) and after the drink (for five minutes). Paired and uunpaired student’s t-test, one-way ANCOVA and one-way repeated measure ANOVA were used for analysis. Blood pressure parameters were followed minute by minute and HRV parameters were compared as a 5-minute of baseline segment to 5-minute post drink segment.

**Results:**

Within-the-group comparison exhibited significant increases in blood pressure parameters (systolic, diastolic, pulse and mean arterial pressure), over a 5-minute post-drinking period in both groups. Zamzam water caused a significant increase in SDRR (an indication of overall HRV) and RMSSD (an indication of vagal activity) as compared to baseline.

**Conclusion:**

Both drinks cause a significant increase in systolic, diastolic, pulse and mean arterial pressure within five minutes post-drinking period. Zamzam water produce a significant increase in cardiac vagal tone but has no effect on cardiac sympathetic activity. Mineral water has no significant effect on both, cardiac vagal and sympathetic activity.

## INTRODUCTION

Simple act of drinking water produces significant transient adjustments in cardiac autonomic control resulting in changes in the heart rate,^1^ and blood pressure.^2^,^3^ A special type of drinkable water is Zamzam water, that is considered “holy water” by Muslims all over the globe. Several analytical studies have been conducted to determine physical/chemical properties and mineral profile of Zamzam water.^4^,^5^ A total of 34 elements have been identified in Zamzam with calcium, magnesium, sodium, and chloride in higher concentrations than natural water. The amount of four toxic elements (arsenic, cadmium, lead, and selenium) was found much below the danger level for human consumption.^6^ Zamzam water differs from ordinary drinking water in terms of its unique radiological, optical, crystallographic and nanotechnological properties, which have been reviewed in a previous study.^4^ Zamzam water is alkaline (average pH is 8) with average lithium, arsenic, and nitrate concentrations of 15 μg/L, 27 μg/L, and 150 mg/L, respectively.^7^

Despite these analytical studies, there is a lack of scientific studies exploring the effects of Zamzam water ingestion on human physiology. To the best of our knowledge, none of the studies have explored the effects of zamzam water on blood pressure (BP) and cardiac autonomic balance/heart rate variability (HRV). Keeping in view the unique radiological, optical, crystallographic, nanotechnological, and analytical properties of Zamzam water, we hypothesize that drinking 500 mL of Zamzam water might elicit a different cardiovascular response in terms of blood pressure and HRV compared to ordinary mineral water.

## METHODS

Students (19-25 years old) registered in the university were invited through advertisements to participate in this study. By considering the mean difference in SDRR (standard deviation of the normal to normal heartbeats) in response to 500 mL mineral water/Zamzam intake (65.05 ± 15.67/77.99 ± 18.89) as obtained from our pilot study, sample size was calculated by OpenEpi version 3.^8^ That gave a sample size of at least 47 subjects per group to have 80% power of detecting this difference at the 5% level of significance.

The Institutional Review Board of the university approved the protocol (Ref. No.: IRB-2014-01-170, dated January 26, 2014) and consent form of this study. The procedures followed were in accordance with the ethical standards of the Helsinki Declaration of 2013.

**Fig.1:**
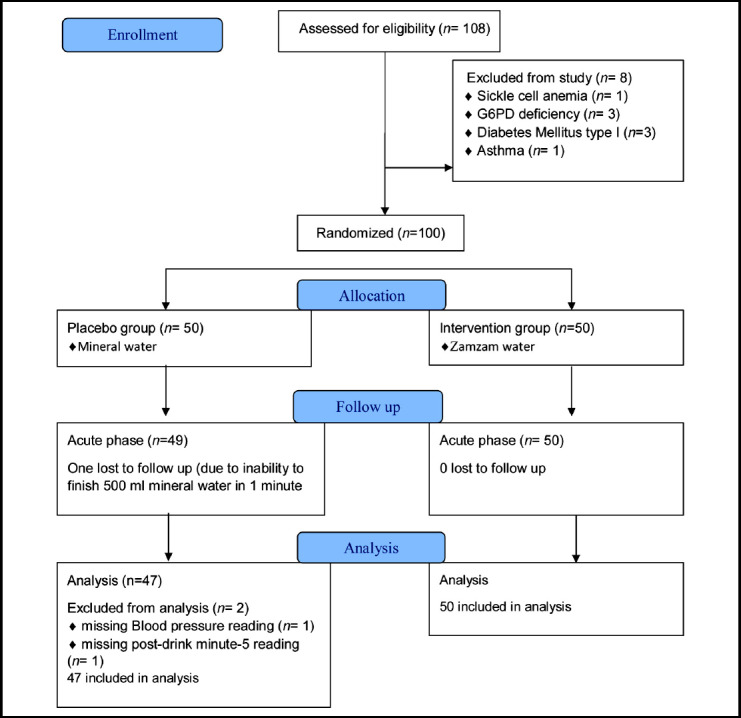
CONSORT Flow Diagram.

The study was a comparative interventional study conducted at the Department of Physiology of Imam Abdulrahman Bin Faisal University in March 2018. Progress through different phases of the study have been shown in Fig.1 with the help of a CONSORT flow diagram. After signing informed consent, subjects were randomly allocated to one of the two groups; one group was given 500 mL of Zamzam, and the other group was given mineral water at room temperature. The dose was selected based on our pilot study results. Randomization of study participants was achieved by a computer-generated random sequence. Both drinks were colorless, odorless, tasteless, and given in similar glasses, without giving study participants or researchers a hint about the identity of the liquid. Subjects were asked to consume the provided quantity (500 mL) in 1-minute. The study participants, researchers recording the ECG and HRV parameters, and statistician analyzing the data, all were blinded to the randomization allocation.

Finometer Pro, device from FMS (Finapress Medical Systems) Netherland was used to obtain continuous finger arterial blood pressure record with a finger pulse transducer. Power Lab system (8/35) with LabChart software (ver 8.1.9), HRV module (ver 2.3) (ADInstruments - Australia), and bioamplifier was used to record electrocardiogram (ECG), with a single limb lead. The electrodes were placed on two shoulders after application of gel and connected to the bioamplifier through the ECG switch box.

### Before the experiment

- Body weight, height, waist and hip circumferences were recorded and body mass index, waist to hip ration and waist to stature ratio were calculated.
- Participants were asked to wear comfortable dress and to arrive at a specified time (since certain parameters of HRV exhibit a circadian pattern; all recordings were done during daytime between 8:00 am to 1:00 pm) with a 12-hour fast on the day assigned to them.

### On the day of the experiment

- The finger blood pressure sensor and ECG leads were properly placed and appropriate adjustments/calibration were done.
- After a relaxation period (5 minutes), baseline values were recorded for five minutes in sitting position. Later, the subjects were provided with 500 mL of either the mineral water or Zamzam water at room temperature and instructed to finish the drink in one minute.
- Further recording was done continuously to get arterial blood pressures; systolic blood pressure (SBP), diastolic blood pressure (DBP), and Mean Arterial Pressure (MAP) and Pulse Pressure (PP) and ECG for the next 5 minutes after liquid intake.
- The whole session took approximately 30 minutes for every participant. The time duration of 5 minutes of ECG recording pre- and post-drink was selected based on the recommendation of the European Society task force and Bourdillon et al.^9^,^10^

### Heart rate variability testing

Lab Chart Pro software (ADInstruments, Australia) was used for data analysis using the HRV module. ECG record was meticulously reviewed for ectopics/artifacts. R waves were sensed by an individually adjusted threshold. HRV was analyzed off-line on the software with HRV module. The relevant HRV parameters of time-domain and frequency-domain were calculated by analyzing the 5-minute recordings pre-and post-drink.

Time-domain parameters were determined by R-R inter-beat intervals and included: mean heart rate (mean HR); mean of the normal to normal heartbeats (mean RR) that reflects an estimate of parasympathetic regulation of the heart; standard deviation of the normal to normal heartbeats (SDRR) that measures overall heart rate variability; and the square root of the mean squared differences of successive RR intervals (RMSSD|), which reflects parasympathetic modulation of the autonomic system.

### The frequency-domain analysis included

1. Low-frequency power (LF), at 0.04–0.15 Hz, involving sympathetic components.
2. High-frequency power (HF), at 0.15–0.4Hz, related to the cardiac parasympathetic or vagal activity (especially vagal efferent activity).
3. The ratio of LF/HF that reflects the sympathovagal balance.

Data was entered in SPSS version 21. Testing for normality of distribution was performed by using Shapiro-Wilk test. Baseline anthropometric parameters between Zamzam and placebo groups were compared by unpaired students’ t-test for parametric data and Mann-Whitney U test for non-parametric data. Blood pressure parameters were followed minute by minute and within-the-groups comparison at different time points (baseline, post-drink minute 1, minute 2, minute 3, minute 4, minute 5) was done by one-way, Repeated measure ANOVA. Where the differences were statistically significant, post-hoc test Bonferroni correction was used. The confidence interval was 95%. HRV parameters were compared as a 5-minute of baseline segment to 5-minute post drink segment by paired and unpaired t test. P< 0.05 value was considered significant for all tests.

## RESULTS

Anthropometric measures, BP, and HRV parameters in two groups were not significantly different at the baseline (Table I, II & III). Between-the-groups comparison (Table-II) showed that intake of 500 mL of either the mineral water or Zamzam resulted in insignificant differences in all four pressure measurements (SBP, DBP, PP and MAP), in every minute of a 5-minute post-drinking period. Within-group comparison by repeated measure ANOVA (Table-II) revealed a significant rise in all four pressure measurements (SBP, DBP, PP and MAP) over a 5-minute post-drinking period.

**Table-I:** Anthropometric parameters of two groups.

	Mineral water group (n:47) (Mean ± SD)	Zamzam group (n:50) (Mean ± SD)	P-value
Age (years)	20.33 ± 0.55	20.44 ± 0.77	0.42
Weight (kg)	60.66 ± 15.77	54.21 ± 16.96	0.06
Height (cm)	159.17 ± 5.00	158.00 ± 6.03	0.30
Waist circumference (cm)	73.99 ± 12.20	71.98 ± 10.21	0.38
Hip circumference (cm)	101.45 ± 13.11	96.89 ± 14.61	0.11
Waist Hip Ratio	0.73 ± 0.05	0.75 ± 0.08	0.16
Waist Stature Ratio	0.47 ± 0.08	0.46 ± 0.06	0.51
Body mass index (kg/m^2^)	23.95 ± 6.27	21.65 ± 6.53	0.08

SD: Standard Deviation.

**Table-II:** Between-the-groups and within-groups comparison of blood pressure parameters.

Study variables	Type of water	Baseline values	Post-drink values					P-value^a^

			Mean minute 1	Mean minute 2	Mean minute 3	Mean minute 4	Mean minute 5	
Systolic blood pressure (mmHg)	Zamzam	124.76 ± 17.15	138.10±15.9*	133.60±14.3*	133.10±14.1*	132.13±13.8*	132.63±13.8*	0.000
	Mineral	123.15 ± 16.83	137.48±16.2*	132.54±15.9*	133.72±15.9*	130.14±15.6*	128.26±14.8*	0.000
	F-statistic	1.96	1.90	2.34	0.17	2.87	0.69	
	P-value^b^	0.87	0.85	0.73	0.84	0.74	0.58	
Diastolic blood pressure (mmHg)	Zamzam	66.90 ± 11.15	73.01±9.4*	71.93±9.8*	71.25±9.9*	71.54±9.5*	71.44±9.5*	0.000
	Mineral	63.55 ± 11.08	70.51±8.8*	69.12±9.2*	69.42±9.3*	69.74±9.6*	70.21±10*	0.000
	F-statistic	1.58	2.56	1.38	4.78	0.96	0.22	
	P-value^b^	0.21	0.18	0.15	0.35	0.36	0.53	
Pulse pressure (mmHg)	Zamzam	57.86 ± 9.85	65.09±11.1*	61.67±9.3*	61.85±9.3*	62.59±9.7*	63.19±9.7*	0.000
	Mineral	59.60 ± 10.57	66.96±11.8*	63.41±11.1*	62.30±11*	63.40±10.8*	62.05±10*	0.000
	F-statistic	2.99	03.78	2.45	0.76	0.72	1.53	
	P-value^b^	0.28	0.42	0.40	0.24	0.18	0.16	
Mean arterial pressure (mmHg)	Zamzam	86.187 ± 12.62	94.70±10.8*	92.49±10.6*	91.87±10.6*	92.4±10.2*	92.50±10*	0.000
	Mineral	83.41 ± 12.30	92.83±10.4*	90.26±10.7*	90.85±10.7*	91.5±10.8*	90.23±11*	0.000
	F-statistic	2.30	0.22	1.98	2.57	0.98	1.67	
	P-value^b^	0.41	0.39	0.31	0.64	0.69	0.90	

P value

a Repeated measure ANOVA; P value

b One-way ANCOVA;

* significant differences as compared to the baseline values at p value < 0.05 by post-hoc test Bonferroni correction.

**Table-III:** Comparison of heart rate variability parameters (5-minute baseline segment vs 5-minute post drink segment).

	Parameters:	Mineral water group (Mean ± SD) (n:47)	Zamzam group (Mean ± SD) (n:50)	P value (between the groups)
1.	Heart Rate (beats/minute)			
	Pre-drink	82.34 ± 9.95	80.55 ± 11.07	0.41
	Post-drink	82.06±9.28	79.96±9.41	0.48
	P value (within the group)	0.80	0.24	
2.	Mean RR (mean of the normal to normal heartbeats) (milliseconds)			
	Pre-drink	721±109	745±110	0.29
	Post-drink	721±110	743±106	0.57
	P value (within the group)	0.97	0.66	
3.	SDRR (milliseconds)			
	Pre-drink	66.34 ± 31.39	60.67 ± 1.93	0.27
	Post-drink	79.06±44.93	73.84±24.37	0.34
	P value (within the group)	0.06	0.00	
4.	RMSSD (milliseconds)			
	Pre-drink	57.25 ± 34.10	54.53 ± 23.01	0.64
	Post-drink	70.62±56.60	74.91±31.44	0.29
	P value (within the group)	0.07	0.02	
5.	LF (normalized unit; nu)			
	Pre-drink	53.02 ± 16.81	48.96± 14.42	0.20
	Post-drink	54.33±17.35	48.64±17.02	0.15
	P value (within the group)	0.62	0.89	
6.	HF (normalized unit; nu)			
	Pre-drink	41.81±14.11	44.66±12.03	0.29
	Post-drink	39.72±13.56	44.84±13.41	0.22
	P value (within the group)	0.28	0.93	
7.	LF/HF			
	Pre-drink	1.62 ± 1.21	1.29 ± 0.80	0.12
	Post-drink	1.79±1.39	1.38±1.16	0.41
	P value (within the group)	0.41	0.56	

**SD:** standard deviation; **SDRR:** standard deviation of the normal to normal heartbeats;

**RMSSD:** square root of the mean squared differences of successive RR intervals;

**LF:** low frequency power; **HF:** high frequency power; Mean Heart Rate: calculated from ECG.

Within-groups comparison of HRV parameters (5-minute baseline segment vs 5-minute post drink segment (Table-III) showed statistically significant increase in RMSSD (an indication of vagal activity) leading to an increase in overall HRV expressed in SDRR, as compared to baseline. However, changes in RMSSD and SDRR were insignificant in mineral water group. Furthermore, there were no significant changes in any of the frequency domain parameters (LF, HF, LF/HF).

It should be noted that the values used in tables were an average for each point in time and not instantaneous values. In addition, the device output data was more in line with showing trends in BP and the BP values obtained through Finometer may not be the actual BP values as recorded through sphygmomanometer.

## DISCUSSION

The aim of the present study was to compare the cardiovascular response in terms of BP and HRV after drinking 500 mL of Zamzam water vs ordinary mineral water. Our study revealed that mineral water and Zamzam were similar in their effects on BP. Both drinks led to a significant increase in SBP, DBP, PP, and MAP (compared to the baseline within the groups), over a 5-minute post-drinking period. Our results agree to the researchers who reported significant pressor effects within 5 minutes of drinking 480 mL tap water in patients with severe autonomic failure.^3^,^11^,^12^ The same pressor response was observed even in the controlled group.^3^

Elicitation of this pressor response within one or few minutes of post-drinking period suggests “sympathetic activation”. In healthy subjects, water drinking has been reported to increase the sympathetic nerve activity in muscles.^13^ The absence of a latent period between water drinking and onset of the pressor response suggests that the increase in the plasma volume or the release of humoral factors like catecholamines, vasopressin, renin etc. has no role in this pressor response.

Statistically significant increase in time domain parameters RMSSD and SDRR in zamzam water group over a 5-minute post-drinking period points towards zamzam-induced increase in cardiac vagal activity. An increase in vagal tone have also been suggested to protect against cardiac arrhythmias and to prolong the life expectancy.^14^ Also, stronger vagal activity has been associated with higher degree of self-regulation,^15^ decreased negative emotional arousal^16^ and better executive cognitive performance.^17^ Hence, future researches should explore the role of Zamzam water in self-regulation, control of negative emotions, and cardiac arrythmias. Furthermore, since the vagus nerve innervates many other organs than the heart, such as the gut, therefore, future research should investigate the effects of zamzam water on vagal tone of other organs as well. Answering those questions may contribute on a theoretical level to elucidate the scientific mechanism of health promoting effects of zamzam water.

### Limitations of the study

It is the short duration of recording following water ingestion. We could not explore that how long the higher cardiac vagal tone is maintained? It would have been great if recording was done for at least half an hour or full one hour. Dosage effect has also not been investigated whereas the recommendation from the Prophet was to drink a lot of Zamzam. Last but not the least, all our subjects were healthy young adults and BP and HRV responses may be buffered in young contrary to the elderly.

## CONCLUSION

Results of the present study exhibit that post-drinking effect of Zamzam water on blood pressure over a period of five minutes are similar to ordinary mineral water. Both drinks cause a significant increase in SBP, DBP, PP and MAP. Regrading HRV parameters, zamzam water produce a significant increase in cardiac vagal tone but has no effect on cardiac sympathetic activity. Mineral water has no significant effect on both; cardiac vagal and sympathetic activity.

### Authors` Contribution

**RL:** Concept and design of study, acquisition of data and analysis, interpretation of data; Drafting the article, final approval of the version to be published.

**FM:** Concept and design of study, acquisition of data and analysis, interpretation of data; revising it critically for important intellectual content, final approval of the version to be published.

**AAS:** Concept and design of study, revising it critically for important intellectual content, final approval of the version to be published.

**RMKAL:** Acquisition of data and analysis, final approval of the version to be published.

**SNAN:** Acquisition of data and analysis, final approval of the version to be published.

All authors are responsible and accountable for the accuracy and integrity of the work.
